# Evaluation of Monoclonal Antibody-Based Sandwich Direct ELISA (MSD-ELISA) for Antigen Detection of Foot-and-Mouth Disease Virus Using Clinical Samples

**DOI:** 10.1371/journal.pone.0094143

**Published:** 2014-04-15

**Authors:** Kazuki Morioka, Katsuhiko Fukai, Kenichi Sakamoto, Kazuo Yoshida, Toru Kanno

**Affiliations:** Exotic Disease Research Station, National Institute of Animal Health, National Agriculture and Food Research Organization, Josuihoncho Kodaira, Tokyo, Japan; The University of Hong Kong, Hong Kong

## Abstract

A monoclonal antibody-based sandwich direct ELISA (MSD-ELISA) method was previously developed for foot-and-mouth disease (FMD) viral antigen detection. Here we evaluated the sensitivity and specificity of two FMD viral antigen detection MSD-ELISAs and compared them with conventional indirect sandwich (IS)-ELISA. The MSD-ELISAs were able to detect the antigen in saliva samples of experimentally-infected pigs for a longer term compared to the IS-ELISA. We also used 178 RT-PCR-positive field samples from cattle and pigs affected by the 2010 type-O FMD outbreak in Japan, and we found that the sensitivities of both MSD-ELISAs were about 7 times higher than that of the IS-ELISA against each sample (P<0.01). In terms of the FMD-positive farm detection rate, the sensitivities of the MSD-ELISAs were about 6 times higher than that of the IS-ELISA against each farm (P<0.01). Although it is necessary to conduct further validation study using the other virus strains, MSD-ELISAs could be appropriate as a method to replace IS-ELISA for FMD antigen detection.

## Introduction

Foot-and-mouth disease (FMD) is caused by the FMD virus (FMDV), a member of the family *Picornaviridae*, genus *Aphthovirus*. FMD is highly contagious and has the economic effect of limiting international trade in livestock and livestock products [1]. FMDV can cause blistering, vesicles and ulcers in the epithelia of the mouth, snout, feet and teat. FMDV consists of seven immunologically distinct serotypes: O, A, C, Asia1, South African Territories (SAT) 1, SAT2 and SAT3. There are some genetically and geographically distinct evolutionary lineages (topotypes) which differ by at least 15% in their VP1 sequences within various serotypes. For example, FMDV type O can be divided into eight topotypes [2]. Antigenic diversity often influences immunoassays for FMDV diagnosis and/or vaccine selection [3], [4]. The FMDV antigenic diagnostic methods mentioned in the World Organization for Animal Health's Office International des Epizooties (OIE) manual [5] are virus isolation, immunological methods—i.e., indirect sandwich–enzyme-linked immunosorbent assays (IS-ELISAs) and the complement fixation test—and nucleic acid detection methods such as reverse transcription-polymerase chain reaction (RT-PCR) and real-time RT-PCR. However, the IS-ELISA is able to do serotyping FMDV, but it does not have sufficient sensitivity [6]–[8].

In an extensive outbreak, it is difficult to collect vesicular fluid and/or vesicular epithelial samples from every suspect farm. In fact, in the 2010 FMD outbreak in Japan, most of the diagnostic samples were oral or nasal swabs, and initial diagnosis was conducted only by RT-PCR for the reason of sensitivity of IS-ELISA which is appropriate for vesicular fluid and/or vesicular epithelial samples. Thus, an antigen-detection ELISA which has high sensitivity enough to detect a viral antigen in samples of saliva and/or nasal discharge must be valuable for the case like 2010 outbreak in Japan.

In our previous study [7], monoclonal antibody (MAb)-based sandwich direct ELISAs (MSD-ELISAs) were developed for multiserotypes (MS) and single serotypes (SS) for FMDV types O, A and Asia1. The MSD-ELISAs were able to detect the different FMDV strains except for MSD-ELISA/SS/Asia1, which showed a weak cross-reaction to type O antigens. In clinical samples, MSD-ELISA/MS and SS/O were able to detect specific FMDV antigens from the saliva and plasma of pigs inoculated with O/TAW/97 (Cathay topotype) [7], and the detection limits of these assays were about 100 to 1000 PFU, as determined by real-time RT-PCR results.

In this study, we evaluated the sensitivity and specificity of the MSD-ELISA reported and compared it with the currently used IS-ELISA, using both experimental samples of other topotypes of serotype O and serotypes A and Asia1 and field samples from the 2010 outbreak of serotype O FMD in Japan.

## Materials and Methods

### Cells and viruses

The virus strains FMDV O/JPN/2000 (ME-SA topotype) [9], [10], O1 BFS 1860 (EURO-SA topotype), A15 TAI 1/60 (ASIA topotype), and Asia1 Shamir ISR were used for animal experiments. Each of these viruses was propagated in the cell lines IBRS-2 [11] and/or BHK-21, which were maintained in Eagle's minimum essential medium containing 5% fetal bovine serum, 0.3 mg/mL of L-glutamine and 1.125 mg/mL of NaHCO_3_, and used as inoculum.

### Ethics statement

Animal experiments carried out in this study were approved by the ethics committee of National Institute of Animal Health, Japan (approval #787, 08–122, 09–029). Field samples used in this study were submitted from Miyazaki prefecture for diagnosis of FMD occurred in 2010 in Japan. These oral and nasal swabs and vesicular epithelial tissues were collected by veterinarians in accordance with the guidelines of Act on Domestic Animal Infectious Diseases Control.

### Laboratory clinical samples

Animal experiments were conducted in a biosafety level 3ag-approved biocontainment facility at our institute. For each virus strains, six or two two-month old pigs were inoculated intradermally with 10^7^ TCID_50_ at the right and front heel bulbs. Saliva samples were collected by cotton swab until 6 days when the clinical signs were definitely observed, and undiluted saliva samples were used for the detection of FMD viral antigens in each assay.

### Field samples

In addition to the samples from animal experiments, a total of 178 RT-PCR-positive samples (135 oral swab samples, 7 nasal samples, 24 oral and nasal swabs soaked in about 10-times volumes of PBS (about 2 ml) and 12 samples of 10% emulsion of homogenized epithelial tissues) collected from cattle and pigs from 78 farms that were affected by the 2010 type O FMD outbreak in Japan caused by O/JPN/2010 (SEA topotype) [12] were used for the comparative studies.

Field samples were submitted from Miyazaki prefecture for diagnosis of FMD occurred in 2010 in Japan. These samples were collected by veterinarians in accordance with the guidelines of Act on Domestic Animal Infectious Diseases Control in which the veterinarian should collect samples such as epithelium or swabs from a lesion and soaked them in 2 ml of PBS.

### Monoclonal antibody-based sandwich direct eLISA for foot-and-mouth disease virus antigen detection

In MSD-ELISAs: the MSD-ELISA for multiserotypes (MS) and the MSD-ELISA for single serotypes (SS) for each serotype (O, A, Asia1), MAb 1H5 (which was produced against O/JPN/2000), which reacts with all seven serotypes of FMDV is used as an antigen-capture-antibody. For the detection of each antigen, the MAbs 1H5, 70C4 (which was produced against O/JPN/2000), 16C6 (which was produced against A15 TAI 1/60), and 12C7 (which was produced against Asia1 Shamir ISR) were used as horseradish-peroxidase (HRPO)-labeled-MAbs for MS, SS for O, A and Asia1, respectively. To improve the specificity of SS for Asia1, MAb 12C7 was used in this study instead of MAb 7C2, which showed slight cross-reaction with the type O strains in previous study. In addition, for the detection of all seven FMDV serotypes in MS, MAb was changed from 71F2 (which was produced against O/JPN/2000) to 1H5. The protocol of the MSD-ELISAs is described in detail in our previous report [7].

### Foot-and-mouth Disease Virus Antigen Detection Indirect Sandwich ELISA

The IS-ELISA by the World Reference Laboratory of FMD was conducted in accord with the OIE manual [5]. The reagents of IS-ELISA (rabbit anti-sera and guinea pig anti-sera) in a lot which we used for this study are as follows: type O (O Taiwan 98 (Cathay topotype)), type A (A 4164 (Asia topotype)), and type Asia1 (Asia1 CAM 9/80).

### Statistics

For analyzing the statistical significance of the differences in virus detection rates between the MSD-ELISAs and IS-ELISA, the Pearson's chi-square test was used.

### RT-PCR

For the RT-PCR for detection of FMDV nucleic acid, the SuperScript III One-Step RT-PCR System with Platinum Taq DNA Polymerase (Invitrogen, Carlsbad, CA) and primers for the 3D region were used [9].

### Real-time RT-PCR

A TaqMan probe and primers for 3D region of FMDV were designed according to the OIE manual [5]. The sequences were as follows: forward primer 5′- ACT GGG TTT TAC AAA CCT GTG A -3′, reverse primer 5′- GCG AGT CCT GCC ACG GA -3′, TaqMan probe 5′-FAM- TCC TTT GCA CGC CGT GGG AC -TAMRA-3′. The program was 48°C for 30 min, 95°C for 10 min, and 40 cycles of 60°C for 15 seconds and 95°C for 1 min. Serial 10-fold dilutions of each FMD virus containing 10^6^ plaque forming unit (PFU)/0.1 ml were used as the positive samples to construct the standard curve.

## Results

### Laboratory clinical samples

Table 1 shows the FMDV antigen detection by the MSD-ELISAs and the IS-ELISA obtained using FMDV (O/JPN/2000, O1 BFS 1860, A15 TAI 1/60 and Asia1 Shamir ISR)-inoculated pig saliva samples. On average, about 0.3 ml of saliva samples were recovered from experimental cotton swabs. In these viruses, O/JPN/2000, A15 TAI 1/60 and Asia1 Shamir ISR are homologous to MAbs used for the MSA-ELISA/SS and heterologous to rabbit and guinea-pig immune sera use in IS-ELISAs. However, O1 BFS 1860 is heterologous antigen for both of the MSD-ELISAs and IS-ELISA. The MSD-ELISAs (especially the MSD-ELISA/SSs) were able to detect each FMDV serotype antigen with high sensitivity and specificity compared to the IS-ELISA. Among the inoculated viruses, the FMDV O/JPN/2000 strain was a low pathogenic virus that showed lower levels of clinical signs compared to the other inoculated FMDV strains (data not shown), and the virus excretion levels of the O/JPN/2000 strain were also lower than those of the other strains (Table 1). Therefore, the IS-ELISA did not show positive results against most of the samples of O/JPN/2000-virus-inoculated pigs. Regarding pigs inoculated with the other FMDV strain (O1 BFS 1860, A15 TAI 1/60 and Asia1 Shamir ISR), the MSD-ELISAs were able to detect FMDV antigens for a longer term compared to the IS-ELISA. The two MSD-ELISAs could detect FMDV antigen at about the same time when the obvious vesicular appeared except for the inoculation site and some samples of inoculated pigs with O1 BFS 1860 and A15 TAI 1/60 showed positive before the vesicular forming. It was generally able to detect about 2 to 3 days after vesicular forming and becoming undetectable with decrease in virus shedding. In all samples, the peak of amounts of detected virus genome (Ct values) and virus antigens (OD values) were almost coincided. The correlation coefficient of the OD values of each ELISA and Ct values are as follows: the MSD-ELISA/MS (*r* = 0.529, *p* = 0.021), the MSD-ELISA/SS (*r* = 0.622, *p* = 0.004) and the IS-ELISA (*r* = 0.31, *p* = 0.240).

**Table 1:** Comparison of the results of FMDV antigen detection methods using saliva of FMDV-inoculated pigs.

Inoculated	Pig			Days post-inoculation						Inoculated	Pig			Days post-inoculation					
virus	no.	Methods*	0	1	2	3	4	5	6	virus	no.	Methods*	0	1	2	3	4	5	6
O/JPN/2000	1	MS	-†	-	-	+	∥+	-	-	A15 TAI 1/60	1	MS	-	+	+	+	-	-	-
		SS	-	-	-	++	+	+	-			SS	-	++	++	++	+	-	-
		IS	-	-	-	-	-	-	-			IS	-	+	+	-	-	-	-
		rPCR	-‡	-	+	++	++	++	+			rPCR	-	+++	+++	++	+	+	+
	2	MS	-	-	-	-	-	+	-		2	MS	-	-	+++	+	+	-	-
		SS	-	-	-	-	+	++	-			SS	-	-	+++	+++	+	-	-
		IS	-	-	-	-	-	-	-			IS	-	-	++	-	-	-	-
		rPCR	-	-	-	+	++	++	+			rPCR	-	+	+++	+++	++	+	+
	3	MS	-	-	-	-	-	-	-		3	MS	-	-	+	+	+	-	-
		SS	-	-	+	-	-	-	-			SS	-	+	++	+++	++	-	-
		IS	-	-	-	-	-	-	-			IS	-	-	-	+	-	-	-
		rPCR	-	-	++	++	++	+	+			rPCR	-	++	++	+++	++	+	+
	4	MS	-	-	-	+	+	-	-		4	MS	-	-	-	+	+	-	-
		SS	-	-	+	+++	+	-	-			SS	-	-	+	++	++	-	-
		IS	-	-	-	-	-	-	-			IS	-	-	-	-	-	-	-
		rPCR	-	-	++	++	++	+	+			rPCR	-	+	++	++	++	+	+
	5	MS	-	-	-	+	-	-	-		5	MS	-	+	+	+	-	+	-
		SS	-	-	+	+	-	-	-			SS	-	++	+++	+++	+	+	-
		IS	-	-	-	-	-	-	-			IS	-	-	+	+	-	-	-
		rPCR	-	-	+	++	+	+	+			rPCR	-	++	+++	+++	++	++	+
	6	MS	-	-	+	-	-	-	-		6	MS	-	+	+	-	-	-	-
		SS	-	-	+++	+	-	-	-			SS	-	++	+++	+++	-	-	-
		IS	-	-	+	-	-	-	-			IS	-	-	+	-	-	-	-
		rPCR	-	-	++	++	+	-	-			rPCR	-	++	+++	++	+	+	+
O1 BFS1860	1	MS	-	+	++	+	+	-	-	Asia1 Shamir	1	MS	-	-	-	+	-	-	-
		SS	-	+++	+++	+++	+	-	-			SS	-	-	+	++	+	-	-
		IS	-	-	-	-	-	+	-			IS	-	-	-	+	-	-	-
		rPCR	-	++	++	++	+	+	-			rPCR	-	-	++	++	++	-	-
	2	MS	-	-	+	+	-	-	-		2	MS	-	-	-	+	+	-	-
		SS	-	+	+++	++	-	-	-			SS	-	-	+	+++	+	-	-
		IS	-	-	-	-	-	-	-			IS	-	-	-	+	-	-	-
		rPCR	-	++	++	++	+	+	-			rPCR	-	+	++	++	++	+	+
	3	MS	-	-	+														
		SS	-	-	+++	§													
		IS	-	-	-														
		rPCR	-	+	++														
	4	MS	-	+															
		SS	-	+++	§														
		IS	-	-															
		rPCR	-	+++															
	5	MS	-	-	++	+++	-	-	-										
		SS	-	+	+++	+++	+	-	-										
		IS	-	+	+	+	-	+	-										
		rPCR	-	+++	++	++	+	+	-										
	6	MS	-	-	++	++													
		SS	-	+	+++	+++	§												
		IS	-	-	+	-													
		rPCR	-	++	++	++													

*MS: MSD-ELISA for multi-serotypes; SS: MSD-ELISA for single serotypes (O, A, Asia1); IS: Indirect sandwich-ELISA for each serotype (O, A, Asia1); rPCR: real-time RT-PCR.

† The OD results (average sample OD-average buffer OD) of the MS, SS and IS ELISAs were as +++, >1.0; ++, 0.5–1.0; +, 0.1–0.5; and −, <0.1.

‡ The results-related plaque-forming unit of rPCR were as +++,>10^4^; ++, 10^2^–10^3^; +, 10^0^–10^2^; and −, <10^0^.

§ The pigs inoculated with virus were euthanized.

∥ Squares mean the day the obvious vesicular appeared except for the inoculated site.

### Field samples

In addition to these samples from animal experiments, we used 178 RT-PCR-positive field samples (135 oral swab samples, 7 nasal samples, 24 oral and nasal swabs soaked in about 10-times volumes of PBS (about 2 ml) and 12 samples of 10% emulsion of homogenized epithelial tissues) from cattle and pigs affected by the 2010 type-O FMD outbreak in Japan to compare the sensitivity of the MSD-ELISAs (MS and SS/O) and IS-ELISA. In the results, the positive sample detection rate of the IS-ELISA was 8.52%, while on the other hand, those of the MSD-ELISA/MS and MSD-ELISA/SS/O were 57.30% and 64.04%, respectively (Table 2). It means that the sensitivities of both MSD-ELISAs were about 7 times higher than that of the IS-ELISA against each sample (P<0.01). However the detection rates of IS-ELISA against oral and/or nasal swabs were low, it seems to depend on the amount of antigen of each sample.

**Table 2:** Sensitivities of the MSD-ELISAs and the IS-ELISA against the FMDV-positive field samples by RT-PCR.

		MSD-ELISA		IS-ELISA
Subject*		MS	SS (type O)	type O
Sample				
	oral swab	56.30%† (76/135)‡	62.50% (85/135)	7.40% (10/133§)
	nasal swab	42.86% (3/7)	57.14% (4/7)	0% (0/7)
	oral/nasal swab	62.50% (15/24)	70.83% (17/24)	4.17% (1/24)
	epithelial tissue	66.67% (8/12)	66.67% (8/12)	33.33% (4/12)
	Total	57.30% (102/178)	64.04% (114/178)	8.52% (15/176)
Farm				
		84.62% (66/78)∥	87.18% (68/78)	14.10% (11/78)

*A total of 178 RT-PCR-positive samples (135 oral swab samples, 7 nasal samples, 24 oral and nasal swab samples, 12 samples of 10% emulsion of homogenized epithelial tissue) collected in the 2010 type O FMD outbreak in Japan from 78 farms were used.

† In both the MSD-ELISAs and the IS-ELISA, OD results ( =  sample OD − average negative OD) of 0.1 or more were judged as positive.

‡ Fractions in parentheses show ELISA-positive samples or farms/RT-PCR-positive samples or farms.

§ The amounts of two samples were insufficient for the test.

∥ The sensitivities against farm units were calculated using the sensitivities against samples.

Based on the sample detection results, we calculated the FMD-positive farm detection rate. In the FMD diagnosis for the FMD free country, if the ELISA showed positive on at least one sample from FMD-suspected farm, it should be regarded as FMD-positive and conduct on immediately stamping-out for control and eradication of the disease. In terms of farm units, the IS-ELISA detected 14.1% of positive farms, and the MSD-ELISA/MS and MSD-ELISA/SS/O detected 84.62% and 87.18% of positive farms, respectively (Table 2). It means that the sensitivities of the MSD-ELISAs were about 6 times higher than that of the IS-ELISA against each farm (P<0.01).

## Discussion

Here we found that the sensitivity of the two MSD-ELISAs against oral and nasal swabs were higher than that of the conventional IS-ELISA. Since RT-PCR is one of the most sensitive diagnostic methods, it was generally used as the primary diagnosis tool for FMD diagnosis in the FMD free countries. However, RT-PCR cannot distinguish serotypes and is at risk for developing contamination. In addition to these reasons, the laboratory diagnosis should be performed by several methods especially for the disease causing severe economic loss for the country, such as FMD. Therefore, an MSD-ELISA could be appropriate as a method to replace IS-ELISA, and it can perform an early serotyping of FMDV using saliva and oral or nasal swabs in which the amount of virus might be low, not to mention epithelial suspensions, vesicular fluids or cell culture supernatants. In this study, we used undiluted saliva samples from inoculated pigs. However, undiluted saliva samples from inoculated cattle generally produced a false negative result, and it could be solved by two times dilution with PBS plus 0.05% Tween 20 (data not shown). Therefore, we considered that there is some inhibition factor against ELISA (antigen–antibody reaction) in saliva of cattle. As for the antigenic matching between viruses and detector antibodies of each ELISA, O/JPN/2010 belongs to SEA topotype, therefore both MSD-ELISA (70C4 originated from O/JPN/2000 (ME-SA topotype)) and IS-ELISA (antisera for Cathay topotype) were heterologous to O/JPN/2010 strain. As a result, we suppose the influence of the antigenic matching between viruses and detector antibodies is not important matter to compare the results of MSD-ELISAs and IS-ELISA in this study.

The MSD-ELISAs could detect FMDV antigen about 2 to 3 days after vesicular forming. Although it was the data from animal experiments, the detectable period would shorter than those of RT-PCR/real-time RT-PCR, also in the field. It is vital for the detection of FMDV antigen by these methods including IS-ELISA that diagnostic samples should be collected from early-stage of disease.

The advantage of using MAbs is to be able to select highly efficient MAbs for high specificity and sensitivity, and uniform affinity to the antigen leads to minimum disparity between the lots compared to polyclonal anti-sera. However, a MAb recognize a single epitope, thus it should be evaluated broad intra- and inter-type reactivity of the MAbs to cover the antigenic variability of FMD viruses. In regard to this point, we have carried out making the panel of our MAbs against recent pandemic FMDV strains in preparation for antigenic varieties (data not shown). Therefore it will be possible to change or combine antigen detection MAbs according to epidemic FMDV strains as needed. To be a lager diagnostic use of the MSD-ELISAs, further validation study should be conducted using field samples of the other virus strains, which are epidemic in especially Asian countries.
