# Correction: Evaluation of Monoclonal Antibody-Based Sandwich Direct ELISA (MSD-ELISA) for Antigen Detection of Foot-and-Mouth Disease Virus Using Clinical Samples

**DOI:** 10.1371/journal.pone.0104052

**Published:** 2014-07-25

**Authors:** The PLOS ONE Staff

Table 1 appears incorrectly due to errors in the typesetting process. The correct version of Table 1 can be viewed below.

**Table 1:** Comparison of the results of FMDV antigen detection methods using saliva of FMDV-inoculated pigs.

Inoculated	Pig			Days post-inoculation						Inoculated	Pig			Days post-inoculation					
virus	no.	Methods*	0	1	2	3	4	5	6	virus	no.	Methods*	0	1	2	3	4	5	6
O/JPN/2000	1	MS	-^†^	-	-	+	^‖‖^ +	-	-	A15 TAI 1/60	1	MS	-	+	+	+	-	-	-
		SS	-	-	-	++	+	+	-			SS	-	++	++	++	+	-	-
		IS	-	-	-	-	-	-	-			IS	-	+	+	-	-	-	-
		rPCR	-^‡^	-	+	++	++	++	+			rPCR	-	+++	+++	++	+	+	+
	2	MS	-	-	-	-	-	+	-		2	MS	-	-	+++	+	+	-	-
		SS	-	-	-	-	+	++	-			SS	-	-	+++	+++	+	-	-
		IS	-	-	-	-	-	-	-			IS	-	-	++	-	-	-	-
		rPCR	-	-	-	+	++	++	+			rPCR	-	+	+++	+++	++	+	+
	3	MS	-	-	-	-	-	-	-		3	MS	-	-	+	+	+	-	-
		SS	-	-	+	-	-	-	-			SS	-	+	++	+++	++	-	-
		IS	-	-	-	-	-	-	-			IS	-	-	-	+	-	-	-
		rPCR	-	-	++	++	++	+	+			rPCR	-	++	++	+++	++	+	+
	4	MS	-	-	-	+	+	-	-		4	MS	-	-	-	+	+	-	-
		SS	-	-	+	+++	+	-	-			SS	-	-	+	++	++	-	-
		IS	-	-	-	-	-	-	-			IS	-	-	-	-	-	-	-
		rPCR	-	-	++	++	++	+	+			rPCR	-	+	++	++	++	+	+
	5	MS	-	-	-	+	-	-	-		5	MS	-	+	+	+	-	+	-
		SS	-	-	+	+	-	-	-			SS	-	++	+++	+++	+	+	-
		IS	-	-	-	-	-	-	-			IS	-	-	+	+	-	-	-
		rPCR	-	-	+	++	+	+	+			rPCR	-	++	+++	+++	++	++	+
	6	MS	-	-	+	-	-	-	-		6	MS	-	+	+	-	-	-	-
		SS	-	-	+++	+	-	-	-			SS	-	++	+++	+++	-	-	-
		IS	-	-	+	-	-	-	-			IS	-	-	+	-	-	-	-
		rPCR	-	-	++	++	+	-	-			rPCR	-	++	+++	++	+	+	+
O1 BFS1860	1	MS	-	+	++	+	+	-	-	Asia1 Shamir	1	MS	-	-	-	+	-	-	-
		SS	-	+++	+++	+++	+	-	-			SS	-	-	+	++	+	-	-
		IS	-	-	-	-	-	+	-			IS	-	-	-	+	-	-	-
		rPCR	-	++	++	++	+	+	-			rPCR	-	-	++	++	++	-	-
	2	MS	-	-	+	+	-	-	-		2	MS	-	-	-	+	+	-	-
		SS	-	+	+++	++	-	-	-			SS	-	-	+	+++	+	-	-
		IS	-	-	-	-	-	-	-			IS	-	-	-	+	-	-	-
		rPCR	-	++	++	++	+	+	-			rPCR	-	+	++	++	++	+	+
	3	MS	-	-	+														
		SS	-	-	+++	^§^													
		IS	-	-	-														
		rPCR	-	+	++														
	4	MS	-	+															
		SS	-	+++	^§^														
		IS	-	-															
		rPCR	-	+++															
	5	MS	-	-	++	+++	-	-	-										
		SS	-	+	+++	+++	+	-	-										
		IS	-	+	+	+	-	+	-										
		rPCR	-	+++	++	++	+	+	-										
	6	MS	-	-	++	++													
		SS	-	+	+++	+++	^§^												
		IS	-	-	+	-													
		rPCR	-	++	++	++													

* MS: MSD-ELISA for multi-serotypes; SS: MSD-ELISA for single serotypes (O, A, Asia1); IS: Indirect sandwich-ELISA for each serotype (O, A, Asia1); rPCR: real-time RT-PCR.

† The OD results (average sample OD-average buffer OD) of the MS, SS and IS ELISAs were as +++, >1.0; ++, 0.51.0; +, 0.10.5; and −, < 0.1.

‡ The results-related plaque-forming unit of rPCR were as +++,<104; ++, 102-103; +, 100-102; and −, >100.

§ The pigs inoculated with virus were euthanized.

‖‖ Squares mean the day the obvious vesicular appeared except for the inoculated site.
